# Hand Grip Strength as a Clinical Biomarker for ME/CFS and Disease Severity

**DOI:** 10.3389/fneur.2018.00992

**Published:** 2018-11-27

**Authors:** Luis Carlos Nacul, Kathleen Mudie, Caroline C. Kingdon, Taane G. Clark, Eliana Mattos Lacerda

**Affiliations:** ^1^Department of Clinical Research, Faculty of Infectious and Tropical Diseases, International Centre for Evidence in Disability, London School of Hygiene and Tropical Medicine, London, United Kingdom; ^2^Department of Infectious Disease Epidemiology, Faculty of Infectious and Tropical Diseases, Faculty of Epidemiology and Population Health, London School of Hygiene and Tropical Medicine, London, United Kingdom

**Keywords:** ME/CFS, fatigue, biomarker, hand grip strength, severity, phenotyping

## Abstract

**Background:** The diagnosis of myalgic encephalomyelitis (ME/CFS) in research and clinical practice has largely relied on clinical history, which can be subjective in nature. Clinical signs are often subtle, overlap with other conditions, and are not formally included as part of diagnostic workup. The characterization of clinical signs and biomarkers is needed for better diagnosis and classification of patients and to monitor treatment response. Hand grip strength (HGS) has been used as an objective measure of muscle strength and fatigue, which is a primary symptom of ME/CFS. We assessed the potential usefulness of HGS as a diagnostic marker in ME/CFS.

**Methods:** We compared HGS measurements from participants in the UK ME/CFS Biobank, with groups consisting of people with ME/CFS of differing severity (*n* = 272), healthy (*n* = 136), multiple sclerosis (*n* = 76) controls, and others with chronic fatigue not meeting the diagnosis of ME/CFS (*n* = 37). We correlated the maximum and minimum of, and differences between, 3 repeated HGS measurements with parameters of disease severity, including fatigue and pain analog scales, and physical and mental component summaries from the SF-36v2TM questionnaire across recruitment groups.

**Results:** HGS indicators were associated with having ME/CFS, with magnitudes of association stronger in severely affected than in mild/moderately affected patients. Compared with healthy controls, being severely affected was associated with a reduction in minimum HGS of 15.3 kg (95%CI 19.3–11.3; *p* < 0.001), while being mild/moderately affected was associated with a 10.5 kg (95%CI 13.2–7.8; *p* < 0.001) reduction. The association persisted after adjusting for age, sex and body mass index. ME/CFS cases also showed lower values of maximum HGS and significant drops in values from the first to second and third trials, compared to other study groups. There were significant correlations between HGS indicators and clinical parameters of disease severity, including fatigue analog scale (Spearman's Rho = −0.40, *p* < 0.001), pain analog scale (Rho = −0.38, *p* < 0.001), and physical component summary (Rho = 0.42, *p* < 0.001).

**Discussion:** HGS is markedly reduced in ME/CFS, particularly in patients with more severe disease, and may indicate muscle and fatigue related symptoms. HGS is a potential diagnostic tool in ME/CFS, and could also be used to enhance patient phenotyping and as an outcome measure following interventions

## Introduction

Fatigue is common in the general population (1–3), and often accompanies infections and chronic disorders of the nervous, cardiovascular, respiratory, musculoskeletal, metabolic, and endocrine systems as well as mood disorders, such as depression and anxiety (4). It also commonly, and temporarily, affects healthy individuals in certain circumstances, such as following periods of excessive or prolonged physical or mental effort, or reduced periods of rest or of good quality sleep.

Myalgic Encephalomyelitis/Chronic Fatigue Syndrome (ME/CFS) presents with disabling symptoms lasting for at least 6 months and resulting in a substantial reduction in activity levels and quality of life (5–7). The etiology is unknown and there are no diagnostic biomarkers for the disease. Prevalence is difficult to determine, ranging from 0.1% to 0.7% (8). Among other symptoms, including post-exertional malaise, unrefreshing sleep, memory, and concentration problems, fatigue is the most recognizable and is considered a central symptom of ME/CFS.

Nevertheless, fatigue may be difficult to characterize and may be confounded by malaise, pain and other issues such as somnolence, dyspnea (difficulty with breathing) and muscle weakness. Patients may use several terms to describe it, such as tiredness, lack of energy or “brain fog,” to represent the difficulty or inability in initiating activity (perception of generalized weakness) (9), reduced capacity in maintaining activity (easy fatigability), and difficulty with concentration, memory and emotional stability (mental fatigue) (10).

The measurement of fatigue in research studies has been subjective and has relied on questionnaires or scales. Symptoms may be exaggerated or underestimated by the individual, and they can vary according to cultural aspects and other factors, such as the presence of other symptoms and mood changes. For these reasons, objective measures of fatigue and disease status are highly desirable, both for diagnostic and classification purposes of people with ME/CFS.

Hand grip strength (HGS) is a reliable measurement of localized muscle strength and reflects the force derived from the combined contraction of extrinsic hand muscles. Originally developed for hand surgery to determine capacity after trauma or surgery, hand grip strength correlates well with other muscle function tests such as knee extension strength (11). Moreover, reduced HGS has been associated with morbidity and mortality, with low values associated with falls, disability, impaired health-related quality of life and prolonged length of stay in hospital (11–13). It has also shown to be strongly correlated with post-operative complications and has been reported as a predictor of loss of functional status and short-term survival in hospitalized patients (14, 15).

In this study, we assess the potential use of HGS parameters as objective measures of disease status and severity in ME/CFS, and correlate it with fatigue and pain severity and with physical and mental functioning.

## Methods

### Study design and population

This was an analytical cross-sectional study using baseline data from participants in the UK ME/CFS Biobank (UKMEB). Participants included people with a medically confirmed ME/CFS diagnosis from the UK National Health Service (NHS) and assessed for compliance with study criteria, i.e., Centers for Disease Control (CDC-94) (6) and/or Canadian Consensus Criteria (CCC) (5); people with apparently normal function and no symptoms of fatigue nor any severe disease (“healthy controls” or “HC”); people with multiple sclerosis (MS) confirmed by an NHS neurologist (“MS cases”); and people with chronic fatigue not compliant with the study criteria (“CF/nonME”).

Procedures for recruitment, selection, and diagnosis have been described previously (16). In summary, participants were recruited through NHS general practices (GPs) and specialist services. All potential UKMEB participants who were aged 18–60 years and gave informed consent were re-assessed by the research team at the recruitment stage for eligibility into the study, which included assessment for compliance with ME/CFS diagnostic criteria for this cohort of participants. The inclusion/exclusion criteria are summarized in Table 1. People with ME/CFS (PWME) were then further stratified by disease severity into two categories: mild/moderately affected (MEmm) if they are ambulatory, and severely affected (MEsa) if they are house- or bed-bound.

**Table 1:** UKMEB inclusion and exclusion criteria.

**Inclusion criteria**	**Exclusion criteria**
ME/CFS cases: clinical diagnosis according to CDC-1994 and/or Canadian Consensus Criteria; diagnosis confirmed by research nurse upon completing baseline assessments	*cases a*nd controls: - recent use (in preceding 3 months) of drugs known to alter immune function, anti-viral medications, and vaccinations - history of acute and chronic infections, such as hepatitis B/C, tuberculosis, HIV, or other severe illness or severe mood disorders - pregnant women and those within 12 months post-partum and/or currently lactating
CF/*non*ME: diagnosis of ME/CFS from clinician but does not fulfill study criteria upon completing baseline assessments	
Healthy controls: no past or present fatiguing and/or other major morbidity, such as cancer or coronary heart disease	
MS cases: confirmed diagnosis made previously by NHS neurologist, in compliance with the NICE guidelines	

*ME/CFS, Myalgic Encephalomyelitis/Chronic Fatigue Syndrome; CF/nonME, chronic fatigue not meeting study criteria for ME/CFS; MS, multiple sclerosis; CDC, Centers for Disease Control; NHS, National Health Services; NICE, National Institute for Health and Care Excellence*.

Ethical approval was granted by the London School of Hygiene & Tropical Medicine (LSHTM) Ethics Committee (ref. 6123), the National Research Ethics Committee (REC; ref. 11/LO/1760, IRAS ID: 77765), and the NHS Research Governance and Developments Offices (R&D), which oversee the recruitment of research participants from government health services.

### Data collection

Data collection ran from March 2012 to December 2015. The study protocol was identical for all participants, regardless of recruitment category.

HGS was quantified during the participant's clinical assessment and examination, by a team member (research nurse or doctor), using a simple precision instrument that offers a quantitative and objective measure of isometric muscular strength of the hand and forearm. We followed standard procedures using a Jamar Hydraulic Hand Dynamometer (model #5030J1–JA Corp) for that aim (17). Participants were seated with back, pelvis, and knees as close to 90 degrees as possible. The shoulder was abducted and neutrally rotated with the elbow flexed at 90 degrees, the forearm neutral, and the wrist held between 0 and 15 degrees of ulnar deviation. The dynamometer was presented vertically, in line with the forearm, to the participant's dominant hand. Grip size was adjusted for comfort (9, 18). Participants were then instructed to squeeze the hand grip as hard as they could, which took ~ 3 s, in three successive trials with 30 s in between each. The entire procedure took ~ 3 min to complete, including instructions.

The strength values were scored using force production in kilograms (0–90). HGS has been shown to have excellent test-retest reliability (intraclass correlations (ICC) 0.97–0.99) and intra-rater reliability (ICC 0.96–0.98) in healthy adults (19) and has been used in various diseases (20–22).

During the clinical assessment, alongside clinical parameters that included height and weight, measures of fatigue and pain intensity were recorded on fatigue (23) and pain (24) analog scales, respectively. The fatigue and pain analog scales are unidimensional measures of intensity and have been widely used in diverse adult populations, e.g., in rheumatic diseases, chronic hepatitis-C infection and systemic lupus (23–26). Each of them can be described as a continuous scale comprised of a horizontal line, 10 centimeters in length, and anchored by two vertical descriptors, one for each symptom extreme (no fatigue/pain and worst imaginable fatigue/pain). High scores, with a maximum of 10, indicate greater intensities of fatigue and pain. Fatigue and pain analog scales have been shown to exhibit good test-retest reliability (*r* = 0.94 for both) and to have high construct validity with 5-point verbal descriptive scales (*r* = 71 and *r* = 0.78, respectfully) (23, 24). Body mass index (BMI) in kg/m^2^ was calculated using participants' height and weight.

In addition, participants completed an extended questionnaire, which includes the SF-36V2^TM^ questionnaire (27), the Fatigue Severity Scale (FSS) (28), and socio-demographic data, such as age and sex, among other variables. The SF-36v2^TM^ comprises of 36 questions providing information on functional status and well-being (29). The answers form eight distinct domains considering physical and mental functions, were summarized into physical (PCS) and mental (MCS) component summary scores. Low scores indicate reduced functional status and reduced mental vitality, respectively. The SF-36v2^TM^ is recognized as a reliable tool that has been used and validated across different populations, and has been used extensively in ME/CFS [L. a. (7, 30, 31)]. A full report of the development of this instrument has been published elsewhere (29). The FSS contains 9 items that relate to statements of fatigue, which are scored between 1 (strongly disagree) and 7 (strongly agree) by the participant. The total score is calculated by adding those attributed to each question, and varies from 9 to 63. Due to the strong correlation between FSS and the fatigue analog scales (*r* = 0.8, *p* < 0.001 in our sample), we opted to use the latter only in our analysis.

### Statistical analysis

Answers to the SF-36v2^TM^ questions were scored in health domains using the SF Health Outcomes^TM^ Scoring Software 4.5 (QualityMetric Inc., RI, United States) and are presented as “normalized” physical and mental summary scores.” Data were analyzed using STATA^TM^ version 15.0 (StataCorp, TX, United States). The maximum and minimum (of three measurements) of HGS were obtained for each participating individual.

Descriptive characteristics were obtained for the whole study population, separated by category of recruitment. Histograms of HGS were visually inspected for shape of distributions. For categorical variables, total numbers and percentages were obtained. For continuous variables, means and standard deviations were provided for normally distributed variables and medians and inter-quartile ranges otherwise. Mean scores (and standard deviations) were calculated for the hand grip strength values. Chi-squared tests and ANOVA F-statistics were used in simple univariate analyses to compare categorical and continuous variables between recruitment categories (32).

To investigate whether HGS was associated with being a ME/CFS case, we plotted minimum and maximum HGS (HGS_min_ and HGS_max_, respectively) against recruitment categories. Bivariate linear regression was used to further explore the associations with indicators of HGS entered into the model as a continuous score and healthy controls (HC) as the baseline comparator. We then adjusted for potential confounding by age, gender, and BMI in multivariate regression analyses.

To examine the change in HGS_min_ and HGS_max_ over the three successive measurements, we plotted their means at each time point within each recruitment category. Differences between HGS indicators (HGS_max_ and HGS_min)_ means at each time point were compared in the following way: time point 1–time point 2, time point 1–time point 3, and time point 2–time point 3.

As the difference between mean HGS at the second and third time points was not statistically different (*p* = 0.21) in the overall study population or in any of the study groups, the average of these two measurements was used for calculating the difference between the first and a subsequent measurement. Therefore, the difference between the first and the average of 2nd and 3rd values represented the overall drop or increase in HGS over subsequent trials, referred to as the HGS-difference (HGS_diff_). Positive values represent a drop in values from the first to subsequent trials. Paired *t*-tests were used to determine whether the means were significantly different within recruitment categories.

To examine whether HGS was correlated with parameters of disease severity, Spearman's rank-order correlations were computed. A correlation matrix was obtained, and graphs produced. To further explore the association of disease severity parameters with indicators of HGS, multivariate regression analyses were performed adjusting for recruitment category, age, sex, and BMI.

## Results

The distributions of participant characteristics by recruitment category are shown in Table 2. Females were over-represented (72%) in the study. Mean age varied across study groups; HC (45.4 years; 95% Confidence Interval (CI) 43.4, 47.4) and CF/nonME (45.4 years; 95%CI 43.4, 47.4) were slightly younger and cases of MS were slightly older (52.5 years; 95%CI 50.6, 54.4). Mean BMI ranged from 24.3 (95%CI 23.0, 25.7) in MEsa to 26.4 (95% ci 25.3, 27.4) in MEmm.

**Table 2:** Characteristics of the study population, separated by recruitment category.

**Factors**		**Healthy Controls (*N* = 136)**	**MS cases (*N* = 76)**	**CF/nonME (*N* = 37)**	**MEmm (*N* = 216)**	**MEsa (*N* = 56)**	*p*-value for difference b/n groups^*^
Sex N(%)	Females	84 (62.8)	59 (77.6)	24 (64.9)	166 (76.9)	43 (76.8)	0.02
Age	mean(SD)	45.4 (12.0)	52.5 (8.4)	45.4 (10.3)	47.1 (11.0)	45.9 (11.5)	0.0001
BMI	mean(SD)	24.9 (4.3)	26.3 (6.1)	24.7 (5.2)	26.4 (6.0)	24.3 (5.0)	0.04
hand grip1	mean(SD)	34.4 (13.9)	23.1 (11.8)	32.2 (15.5)	25.2 (11.9)	21.2 (9.7)	< 0.0001
hand grip2	mean(SD)	33.9 (14.0)	22.5 (12.9)	31.4 (17.2)	23.5 (12.6)	19.2 (9.2)	< 0.0001
hand grip3	mean(SD)	34.1 (14.2)	22.6 (12.7)	31.4 (18.1)	23.9 (12.4)	18.5 (9.2)	< 0.0001
min hand grip	mean(SD)	32.0 (13.8)	20.3 (12.0)	29.0 (16.8)	21.6 (11.9)	16.7 (9.2)	< 0.0001
max hand grip	mean(SD)	36.2 (14.1)	25.1 (12.4)	34.2 (16.8)	27.1 (12.2)	22.9 (9.2)	< 0.0001
Fatigue Analog Scale	mean(SD)	1.5 (1.5)	5.3 (2.5)	4.5 (2.2)	6.7 (1.6)	7.4 (1.4)	< 0.0001
Pain Analog Scale	mean(SD)	1.0 (1.5)	3.4 (2.7)	2.2 (2.0)	4.9 (2.5)	5.3 (2.7)	< 0.0001
PCS	mean(SD)	57.0 (4.9)	38.4 (12.2)	45.7 (8.4)	31.0 (8.6)	19.0 (4.7)	< 0.0001
MCS	mean(SD)	52.1 (8.1)	46.1 (10.8)	43.7 (9.0)	39.2 (9.9)	44.2 (9.9)	< 0.0001

* *χ^2^ for categorical variables; F-statistic for continuous variables. HC, healthy controls; MS, multiple sclerosis; CF/nonME, chronic fatigue not meeting study criteria for ME/CFS; MEmm, ME/CFS mild/moderately affected; MEsa, ME/CFS severely affected; BMI, body mass index; PCS, physical component summary; MCS, mental component summary; SD, standard deviation*.

The mean values for the fatigue analog scales were 7.4 (95%CI 7.0, 7.7) for MEsa and 6.7 (95%CI 6.4, 7.0) for MEmm. These values were significantly (*p* < 0.0001) higher than for HC (1.5; 95%CI 1.2, 1.8), CF/nonME (4.5; 95%CI 3.4, 5.5) and MS cases (5.4; 95%CI 4.7, 5.9). The pain analog scales were also significantly higher in ME/CFS cases (*P* < 0.0001); the mean values were: 5.3 (95%CI 4.6, 6.0) for Mesa, 4.9 (95%CI 4.6, 5.3) for MEmm, and 1.0 (95%CI 0.7, 1.2) for HC, with values for other groups in between these. The Physical Component Summary (PCS) in particular, and also Mental Component Summary (MCS) scores were much lower (*p* < 0.001) in MEsa, (19.0 PCS; 95%CI 17.7, 20.4 and 44.2 MCS; 95%CI 41.4, 46.9) and MEmm (31.0 PCS; 95%CI 30.0, 32.2 and 39.2 MCS; 95%CI 37.8, 40.6) compared with HC (57.0 PCS; 95%CI 56.2, 57.9 and 52.1 MCS; 95%CI 50.7, 53.5), CF/nonME (45.7 PCS; 95%CI 42.9, 48.6 and 43.7 MCS; 95%CI 40.6, 46.8) and MS cases (38.4 PCS; 95%CI 35.6, 41.2 and 46.1 MCS; 95%CI 43.6, 48.5), indicating reduced functional status and mental vitality among people with ME/CFS (Table 2).

**Figure 1:**
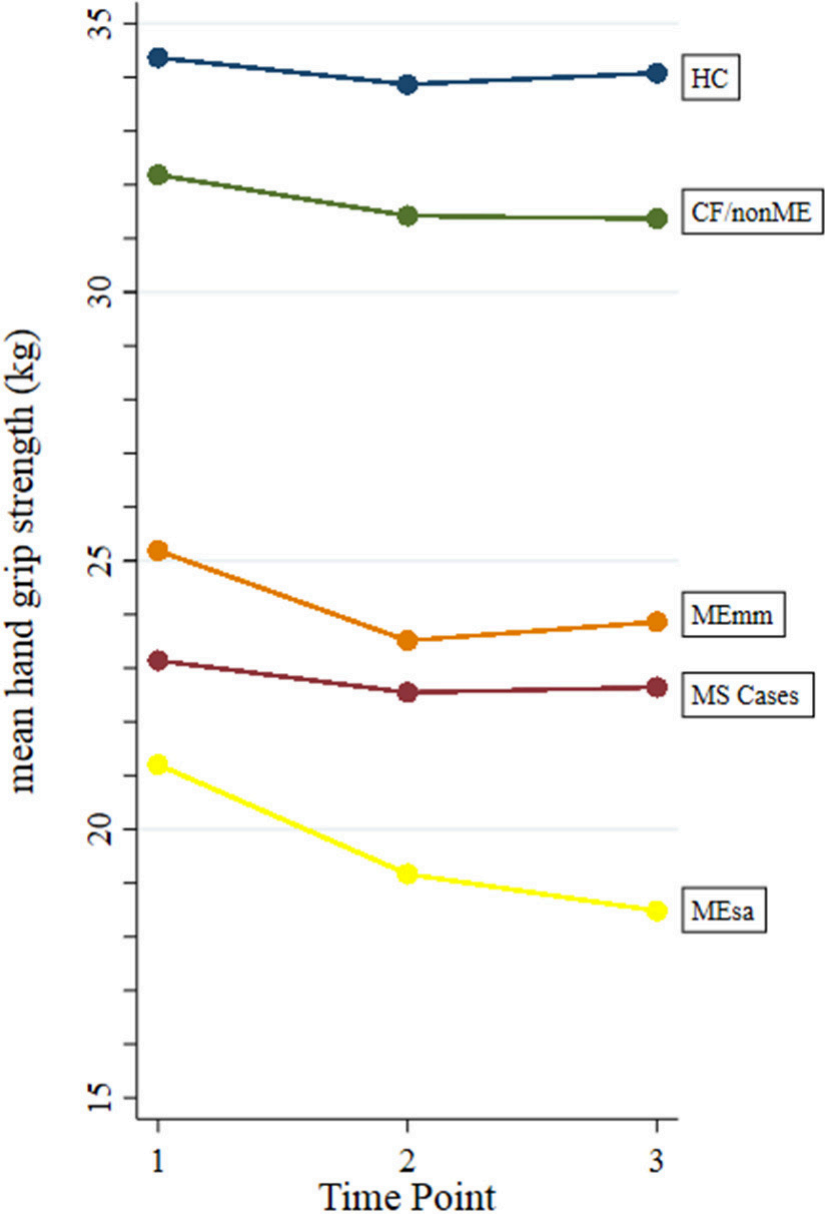
Mean hand grip strength at each time point within each recruitment category. HC, healthy controls; MS, multiple sclerosis; CF/nonME, chronic fatigue not meeting study criteria for ME/CFS; MEmm, ME/CFS mild/moderately affected; MEsa, ME/CFS severely affected.

When mean values of HGS were observed over time (i.e., over successive trials), no trend was seen within HC, MS cases, and CF/nonME (Figure 1). Among these recruitment categories, there was a slight (non-significant) drop in values between the first and second trials, which was typically followed by a slight increase in values from the second to the third trials. A similar trend was found for MEmm, except that the drop in values between the 1st and 2nd trials was more marked (*p* < 0.01). However, among MEsa, the mean HGS decreased markedly from first, to second (*P* = 0.03), and then again to the third time point (*P* = 0.13), whereas HGS for MEmm increased from second to third trial (*P* = 0.19).

### Associations of hand grip strength parameters with being a ME/CFS case (HGS_max_, HGS_min_, and HGS_diff_)

**Figure 2:**
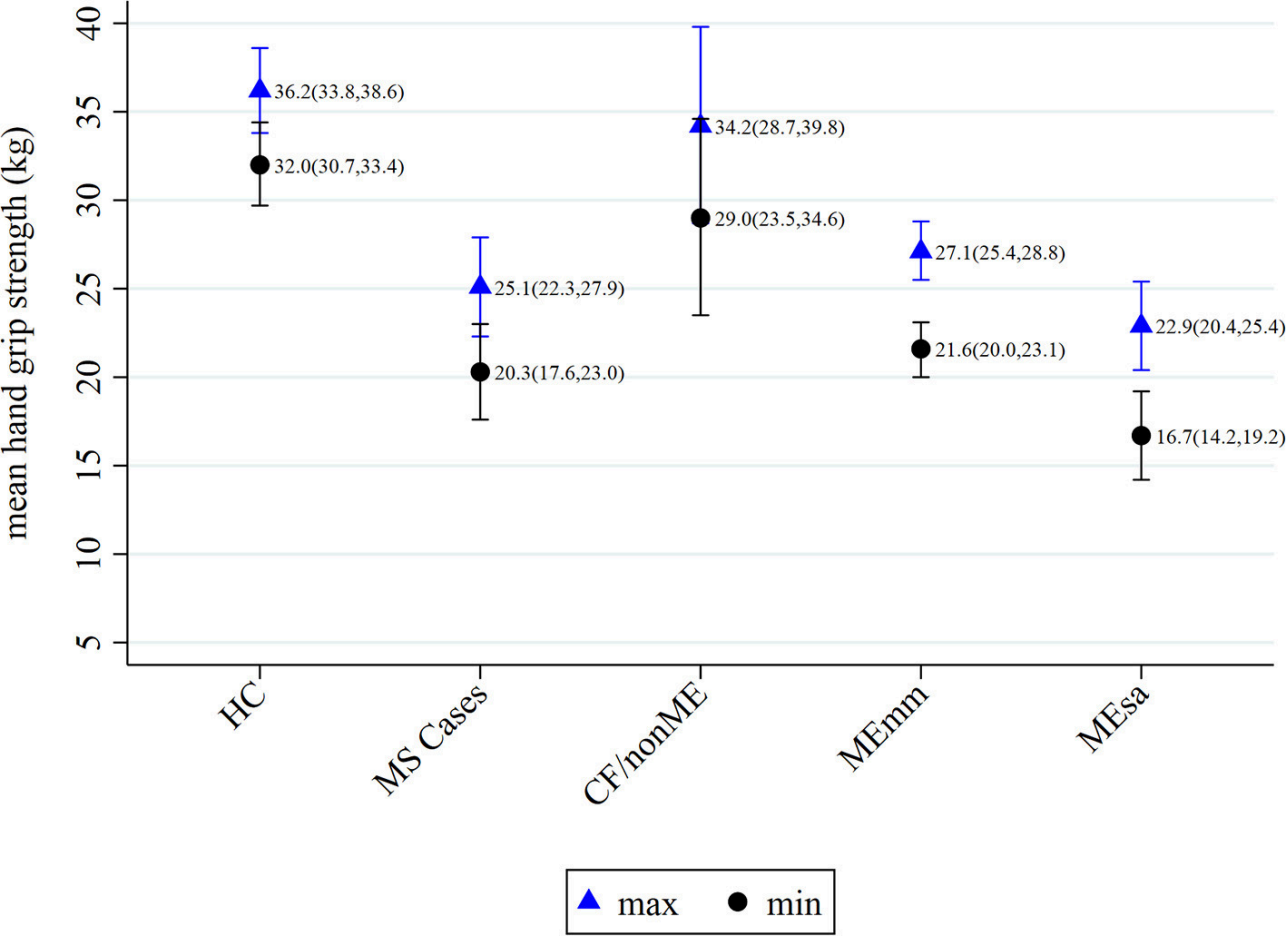
Means of maximum and minimum hand grip strengths within recruitment categories, with 95% confidence intervals. HC, healthy controls; MS, multiple sclerosis; CF/nonME, chronic fatigue not meeting study criteria for ME/CFS; MEmm, ME/CFS mild/moderately affected; MEsa, ME/CFS severely affected.

The mean of the HGS_max_ measurements was highest among HC (36.2 kg; 95%CI 33.8, 38.6) and lowest among MEsa (22.9 kg; 95%CI 20.4, 25.4) (*p* < 0.0001). The same was true for the average HGS_min_ measurements, with HC producing a mean of 32.0 kg (95%CI 30.7, 33.4) and MEsa,16.7 kg (95%CI 14.2, 19.2). Among HC, MS and CF/nonME cases, both the HGS_max_ and HGS_min_ values were similar (Figure 2).

Table 3 shows the difference between HGS parameters of various study groups and HCs, with negative values indicating values below that of HCs. Compared with HCs, the values of HG_min_ were on average 15.3 kg lower in MEsa (−15.3 Kg; 95%CI −19.3, −11.3), 11.8 kg lower in MS cases (−11.8 kg; 95%CI −15.3, −8.2), and 10.5 kg lower in MEmm cases (−10.5 kg; −13.2, −7.8). These differences were all statistically significant at *P* < 0.001. CF/nonME values were similar to that of HCs (−3.0; 95%CI −7.6, 1.5; *P* = 0.19). The same trend was found for HGS_max_ but was less pronounced. After adjusting for age, sex, and BMI, changes in mean HGS compared with HC were attenuated for all but CF/nonME. MEsa still showed the lowest HGS_min_ value (−10.2 kg; 95%CI −13.3, −7.1) compared with HC, however MS cases (−5.9 kg; 95%CI −8.8, −2.9) now showed similar values to CF/nonME cases (−5.5; 95%CI −10.0, −1.0).

**Table 3:** Crude and adjusted associations of minimum and maximum hand grip strengths with recruitment categories, compared with healthy controls using ANOVA.

	**Crude**		Adjusted^*^	
**Factors**	**Change in mean HGS kg (95%CI)**	*p*-value^**^	**Change in mean HGS kg (95%CI)**	*p*-value^**^
**HGS**_min_				
HC	0.0		0.0
MS controls	−11.8 (−15.3, −8.2)	< 0.001	−5.9 (−8.8, −2.9)	< 0.001
CF/nonME	−3.0 (−7.6, 1.5)	0.19	−5.5 (−10.0, −1.0)	0.02
MEmm	−10.5 (−13.2, −7.8)	< 0.001	−7.6 (−10.1, −5.1)	< 0.001
MEsa	−15.3 (−19.3, −11.3)	< 0.001	−10.2 (−13.3, −7.1)	< 0.001
**HGS**_max_				
HC	0.0		0.0
MS controls	−11.0 (−14.7, −7.4)	< 0.001	−5.3 (−8.2, −2.4)	< 0.001
CF/nonME	−2.0 (−6.6, 2.7)	0.41	−4.3 (−8.8, 0.24)	0.06
MEmm	−9.1 (−11.8, −6.3)	< 0.001	−6.1 (−8.6, −3.5)	< 0.001
MEsa	−13.3 (−17.3, −9.2)	< 0.001	−7.9 (−11.1, −4.8)	< 0.001

* adjusted for sex, age, and BMI

** *t-statistic. HC, healthy controls; MS, multiple sclerosis; CF/nonME, chronic fatigue not meeting study criteria for ME/CFS; MEmm, ME/CFS mild/moderately affected; MEsa, ME/CFS severely affected; BMI, body mass index*.

The results for the HGS_diff_ are shown in Table 4. Overall, for all recruitment categories, there was a slight decrease in mean HGS_diff_ (1.07 kg; 95%CI 0.68, 1.47; *p* < 0.001). For HC (0.39 kg; 95%CI −0.20, 0.99 *p* = 0.19), MS cases (0.64; 95%CI −0.32, 1.60; *p* = 0.19), and CF/nonME (0.79; 95%CI −0.74, 2.32; *p* = 0.30), none of these differences were significant. However, for ME/CFS cases (both MEmm and MEsa), the HGS_diff_ were higher and statically significant (*P* < 0.01).

**Table 4:** Comparison of difference between hand grip strength at time point 1 and the average of hand grip strength at time points 2 and 3 within each recruitment category, using paired t-test.

	**Paired Differences (hand grip 1–avg hand grip 2 and 3)**			
		**95% CI**	
**Pairs**	**Mean**	**Lower**	**Upper**	***p*-value**
Overall	1.07	0.68	1.47	< 0.001
HC	0.39	−0.20	0.99	0.19
MS cases	0.64	−0.32	1.60	0.19
CF/nonME	0.79	−0.74	2.32	0.30
MEmm	1.38	0.75	2.01	< 0.0001
MEsa	2.38	0.54	4.22	0.01

*HC, healthy controls; MS, multiple sclerosis; CF/nonME, chronic fatigue not meeting study criteria for ME/CFS; MEmm, ME/CFS mild/moderately affected; MEsa, ME/CFS severely affected*.

### Correlations of hand grip strength with parameters of disease severity

Overall, HGS_max_ and HGS_min_ were low to moderately correlated with clinical parameters of disease severity, including fatigue and pain analog scales and PCS, but weakly correlated with MCS (Supplementary Table 1).

Results from bivariate and multivariate regression analyses for the association of HGS indicators and parameters of disease severity are presented in Table 5. For every one unit increase in kilograms of HGS_min_, fatigue severity and pain severity analog scales decreased by 0.23 (95%CI −0.29, −0.17) and 1.47 (95%CI −1.86, −1.08), respectively. Alternatively, PCS increased by 0.33 kg (95%CI 0.26, 0.41) and MCS increased by 0.21 kg (95%CI 0.10, 0.32) for each unit increase in kilograms of HGS_min_. The same can be seen for HGS_max_, but less markedly. Adjustment for the variables age, sex, and BMI resulted in slightly weaker, but still significant, associations in all cases.

**Table 5:** Crude and adjusted associations of hand grip strength indicators with disease severity parameters.

**Disease severity parameters**	**Crude**		Adjusted^*^	
	**Change in mean hand grip strength kg (95%CI)**	*p*-value^**^	**Change in mean hand grip strength kg (95%CI)**	*p*-value^**^
**HGS**_min_				
Fatigue analog scale	−0.23 (−0.29, −0.17)	< 0.001	−0.14 (−0.20, −0.08)	< 0.001
Pain analog scale	−1.47 (−1.86, −1.08)	< 0.001	−0.93 (−0.17, −0.69)	< 0.001
PCS	0.33 (0.26, 0.41)	< 0.001	0.24 (0.17, 0.31)	< 0.001
MCS	0.21 (0.10, 0.32)	< 0.001	0.07 (0.01, 0.13)	0.02
**HGS**_max_				
Fatigue analog scale	−0.20 (−0.26, −0.13)	< 0.001	−0.13 (−0.19, −0.07)	< 0.001
Pain analog scale	−1.29 (−1.68, −0.89)	< 0.001	−0.84 (−1.07, −0.60)	< 0.001
PCS	0.30 (0.22, 0.37)	< 0.001	0.24 (0.18, 0.31)	< 0.001
MCS	0.17 (0.06, 0.28)	< 0.001	0.07 (0.01, 0.13)	0.03

* *adjusted for recruitment category, age, sex, and BMI*;

** *t-statistic. PCS, physical component summary; MCS, mental component summary; BMI, body mass index*.

## Discussion

### Concepts of fatigue, strength, physical functioning, and their measurement

The concept of fatigue is multidimensional and lacks a universally accepted definition. It may be central or peripheral in origin. Central fatigue refers to a state of less-than-optimal outputs from the brain, in particular, from cortical motor area to motor units where nervous fibers stimulate muscle fibers to produce contraction. In contrast, peripheral fatigue represents an impairment of the contractile function of skeletal muscle fibers and the inability of the muscle to produce force (33, 34).

In ME/CFS, fatigue is a key symptom, used for the diagnosis and the assessment of disease severity. However, there is no single descriptor that accurately defines it. It is usually assessed by direct questioning and reported presence of the symptom during diagnosis workup; symptom classifiers may include duration, frequency, persistence or recurrence, and intensity. When establishing compliance with diagnostic criteria, people may be asked, for example, how long they have experienced fatigue, and whether it is present for more than 50% of the time.

Questionnaires, such as the UKMEB symptoms assessment or clinical phenotyping questionnaires (16), or the DePaul Symptoms Questionnaire (35) have been used to establish the presence and severity of fatigue and other symptoms. The fatigue (23) and pain analog scales (24), which are used in this study, are simple and widely used instruments to ascertain fatigue and pain severity; they do, however, rely on self-reporting. This undoubtedly carries some subjectivity and, although both internal validity and test-retest reliability have been shown to be high (24), it is more difficult to establish comparability in the way different individuals interpret and report on fatigue and its severity. This may be particularly problematic in the case of ME/CFS, where the experience of fatigue is usually both physical and mental–described as “lack of stamina or physical energy” and “brain fog and cognitive problems,” respectively—and is closely associated with a range of other symptoms. Such symptoms may or may not be interpreted as part of the same symptom complex, which may include post-exertional malaise, pain, flu-like symptoms and unrefreshing sleep, to name a few associated symptoms. The pathological fatigue experienced by people with ME/CFS, which some refer to as “ME fatigue”, to distinguish from fatigue or tiredness that represent everyday experience, may be very hard to express and quantify in objective terms.

The experience of fatigue or of feeling ill (with ME/CFS) may also be measured indirectly through the impact on people's lives, such as on the ability of individuals to perform physical or mental tasks, including self-care or engaging in work, study and social activities. Some fatigue scales incorporate the impact of fatigue on functioning (28), but more generally, instruments that measure functionality or quality of life have been used to indicate the impact of the health status on individuals affected. The SF-36v2^TM^ is one such well-validated and widely used instrument, and we used in our analyses the Physical and Mental Component Summaries derived from answers given by participants, as proxy measures for the impact of fatigue and disease on the life of individuals studied.

With the challenges involved in measuring fatigue, and more broadly disease severity in ME/CFS, the importance of an objective measurement cannot be overestimated, particularly one which could be used in research studies to aid diagnosis and clinical phenotyping. Assessments indicating levels of severity and impact could be used on a longitudinal basis to inform disease progress and, potentially, disease prognosis.

### Hand grip strength as a tool for measuring disease status

Although the testing of HGS was originally created to evaluate patients undergoing hand surgery, this measurement has been shown to be associated with reduced muscle strength and decreased physical fitness more broadly (36). The latter is one of the strongest predictors of individual future health status, characterized by the ability to perform daily activities with vigor and without overdue fatigue. Physical fitness is an important predictor of mortality and morbidity for older and younger adults and teenagers, which can be applied in socially, economically and culturally diverse populations (36–39).

Reduced muscle strength and decreased hand grip have been associated with a few specific situations, such as muscle or nerve injury and malnutrition. More broadly, though, grip strength has been shown to be a simple, yet powerful indicator of overall physical health status and as a predictor of future disability, morbidity, health deterioration (40) and mortality (15), and to assess treatment in various diseases, such as chronic obstructive pulmonary disease (41) and rheumatoid arthritis (20, 42). HGS has also been used to predict cardiovascular risk in pre-diabetic and diabetic patients (38). Associations of poor HGS and future disability and mortality have been observed even among healthy subjects (43), suggesting it could perhaps be used as an early, though nonspecific, indicator of risk for health deterioration.

The underlying mechanisms explaining the association between grip strength and health status are poorly understood, except in cases where local factors such as upper limb muscle damage are in place. Nevertheless, there is sufficient evidence that HGS is a measurement of not only muscle strength, but also of overall physical health. However, unlike cardiorespiratory fitness testing, which demands special location and equipment, measurement of HGS is a simple and mobile tool; making them particularly useful for community-based health evaluations, especially for severe cases of ME/CFS, who normally are house-bound.

### Summary of results and interpretation

Overall, patients with ME/CFS and MS had significant lower HGS values than HC. MS is the most common immune-mediated inflammatory demyelinating disease of the central nervous system. One of the most prominent features of MS is motor weakness. Therefore, it is expected people with MS to display lower HGS (44, 45). However, it is interesting that ME/CFS patients also had significantly lower HGS values compared to HC, even after controlling for age, sex, and BMI, with even mild cases showing lower HGS. People with ME/CFS are not malnourished and have preserved muscle tonus, suggesting that other than local factors related to the integrity of upper limb must be involved. We suggest these might relate to ongoing inflammation or disruption of signaling mechanisms between central nervous system and periphery, and it may also represent an overall measurement of “physical health and functioning.”

Furthermore, HGS among people with ME/CFS significantly dropped in measured strength between the first and subsequent trials, when compared with HC. This effect was not observed in cases of MS nor in those with chronic fatigue which did not meet the criteria for ME/CFS. This finding may relate to early fatigability, where an already reduced ability to produce substantive muscle power in the first trial is further compromised in subsequent attempts. The understanding of the mechanisms behind the lack of rapid recovery in demonstrable muscle force produced between subsequent (hand grip) trials, may be the key to explaining the pathological nature of fatigability and post-exertional symptoms in people with ME/CFS. It is possible that disruptions in muscle energy metabolism or in the continuous production and release of energy by muscle cells, or in nervous system signaling could be involved, however, further experiments would be required for any conclusions to be made.

We have also shown that higher HGS was associated with lower fatigue and pain intensities and with higher functional status and mental vitality. The correlations were stronger for physical than mental component summaries of the SF-36v2^TM^, suggesting a lesser role for lack of motivation as a single factor explaining poorer results in those with ME/CFS. Such significant correlations of HGS values and indicators of symptom severity and disease status provide further indications for the value of including of HGS as an objective test to enhance patient phenotyping in ME/CFS as part of clinical practice and in research.

Our results are in line with previous small studies, and reinforce the importance of HGS as part of the clinical assessment of people with ME/CFS (46). Patients meeting the CDC-94 criteria for ME/CFS (6) had previously shown significantly reduced HGS_max_ compared to non-fatigued individuals, with example values for right hand force of 31 Kg in ME/CFS vs. 42 Kg in healthy sedentary controls (*n* = 8 in each group) (47) and 24.3 Kg in ME/CFS vs. 35.8 Kg in healthy controls (*n* = 30 and 15 in CFS and controls, respectively) (48). However, no difference in values was found comparing PWME to those with major depression (48). HGS was also used to assess the effects of an exercise intervention among 11 women with ME/CFS meeting either CDC-1994 (6) or International Consensus Criteria [B. M. (49)], who showed a significant improvement in left hand HGS (from 20 to 26 Kg), but not in right hand HGS following the intervention (50), suggesting a role for HGS as an outcome measure in the evaluation of interventions. People with ME/CFS were also previously shown to have slower and incomplete recovery of HGS values following effort challenge, compared with non-fatigued (51, 52) and controls with MS (51). These studies included 48 and 10 ME/CFS cases, respectively.

### Study strengths and limitations

The study included a relatively large number of participants with ME/CFS (*N* = 272), including different levels of severity, and used both healthy and diseased individuals for comparison groups. We used standardized methods for diagnosis and characterization of participants, which included rigorous procedures for selection, clinical assessment and phenotyping, according to the UK ME/CFS Biobank protocol (16). This was, however, an observational cross-sectional study, and the use of HGS as a diagnostic tool and the mechanisms by which variation in values reflect pathophysiology will require further studies. Similarly, validation of the study in individuals with a range of disease durations, including those with more recent disease as well as in different geographical locations and ethnicities, will be needed to widen the representativeness of the study to other populations and in patients at various disease stages. Furthermore, there are multiple types of MS, and by combining cases of MS all into one category, the results may have been diluted. However, this is not likely to have made a significant difference as differences in dynamic fatigability have been found when comparing MS and healthy controls but not when comparing types of MS (45).

## Conclusions and implications

In this study, we investigated the potential use of HGS as an objective measure of disease status and severity in people with ME/CFS and assessed the correlation of HGS with fatigue/pain severity and physical/mental functioning. HGS was markedly reduced in people with ME/CFS, particularly in those who were severely affected. Furthermore, strength decreased with each successive measurement among people with ME/CFS, which suggests early fatigability, or that they tire more easily than healthy or diseased controls. The abnormal pattern of handgrip strength shown in ME/CFS cases give further indications of the distinct nature of ME/CFS and shed more light into the pathological nature of the fatigue symptom complex experienced by those with the disease. The exact mechanisms involved in reduced power and fatigability require further exploration. Nevertheless, the results shown here have practical implications in better defining a fatigue phenotype that help identify cases of ME/CFS and that can be used as an objective tool for diagnosis and measuring disease severity.

## Author contributions

LN conceived, and with KM, CK, and EL, designed and conducted the study and acquired the data. LN and KM designed the analyses and interpreted the data. All authors contributed to drafting and to revising the manuscript critically for important intellectual content. All authors approved the final version of the manuscript to be published.

### Conflict of interest statement

The authors declare that the research was conducted in the absence of any commercial or financial relationships that could be construed as a potential conflict of interest.
