# Supplementary material for: Hand Grip Strength as a Clinical Biomarker for ME/CFS and Disease Severity
Source: Front Neurol. 2018 Nov 27;9:992. doi: 10.3389/fneur.2018.00992 (PMC6277492; doi:10.3389/fneur.2018.00992)
Supplement: Supplementary file 1 [file Table_1.DOCX]

**Supplementary Materials**

Table 1: Correlations of minimum and maximum hand grip strength with parameters of disease severity, including fatigue and pain analog scales and physical and mental component summaries of SF-36v2^TM^, using Spearman's rank-order correlation*

|  | **Minimum hand grip** | **Maximum hand grip** | **Fatigue analog scale** | **Pain analog scale** | **PCS** | **MCS** |
| --- | --- | --- | --- | --- | --- | --- |
| Minimum hand grip | 1.0 |  |  |  |  |  |
| Maximum hand grip | 0.94* | 1.0 |  |  |  |  |
| Fatigue analog scale | -0.40* | -0.35* | 1.0 |  |  |  |
| Pain analog scale | -0.38* | -0.33* | 0.71* | 1.0 |  |  |
| PCS | 0.42* | 0.36* | -0.75* | -0.72* | 1.0 |  |
| MCS | 0.22* | 0.18* | -0.53* | -0.43* | 0.29* | 1.0 |

*p<0.0001. PCS: physical component summary; MCS: mental component summary.
